# A new regulation mechanism for KCNN4, the Ca^2+^-dependent K^+^ channel, by molecular interactions with the Ca^2+^pump PMCA4b

**DOI:** 10.1016/j.jbc.2024.108114

**Published:** 2024-12-21

**Authors:** Benoit Allegrini, Morgane Mignotet, Raphaël Rapetti-Mauss, Franck Borgese, Olivier Soriani, Hélène Guizouarn

**Affiliations:** 1 Université Côte d’Azur, CNRS, Inserm, Institut Biologie Valrose, Nice, France; 2 Laboratory of Excellence for RBC, LABEX GR-Ex, Paris, France

**Keywords:** KCNN4, red blood cell, calcium pump, PMCA, KCNN2

## Abstract

KCNN4, a Ca^2+^-activated K^+^ channel, is involved in various physiological and pathological processes. It is essential for epithelial transport, immune system, and other physiological mechanisms, but its activation is also involved in cancer pathophysiology as well as red blood cell (RBC) disorders. The activation of KCNN4 in RBC leads to loss of KCl and water, a mechanism known as the “Gardos effect” described 70 years ago. This Ca^2+^-induced dehydration is irreversible in human RBC and must be tightly controlled to prevent not only hemolysis but also alterations in RBC rheological properties. In this study, we have investigated the regulation of KCNN4 activity after changes in RBC Ca^2+^ concentration. Using electrophysiology, immunoprecipitation, and proximity ligation assay in human embryonic kidney 293–transfected cells, K562 cells, or RBCs, we have found that KCNN4 and the Ca^2+^ pump PMCA4b (plasma membrane calcium-transporting ATPase 4b) interact tightly with each other, such that the C-terminal domain of PMCA4b regulates KCNN4 activity, independently of the Ca^2+^ extrusion activity of the pump. This regulation was not restricted to KCNN4: the small-conductance Ca^2+^-activated K^+^ channel KCNN2 was similarly regulated by the calcium pump. We propose a new mechanism that could control KCNN4 activity by a molecular inhibitory interaction with PMCA4b. It is suggested that this mechanism could attenuate erythrocyte dehydration in response to an increase in intracellular Ca^2+^.

KCNN4 is one of the four members of the K^+^ channel family exclusively activated by intracellular Ca^2+^. It was initially discovered in red blood cells (RBCs) where the Ca^2+^-induced K^+^ loss leading to cell dehydration was named the “Gardos effect” after its Hungarian discoverer (1). Later on, K^+^ channels of intermediate conductance that are activated by the increase in cytoplasmic Ca^2+^ have been discovered in various cell types. This conductance is referred to by several names such as IKCa1, IK1, SK4, K_Ca_3.1, and Gardos channel for instance. The channel was first cloned in human pancreas cells (2), but it is expressed in many other cell lines. By regulating membrane potential and Ca^2+^ signaling, it is a key player in immune system, epithelial transport, and cardiac activity (3, 4, 5, 6, 7, 8).

In RBC, the activation of KCNN4 by high intracellular Ca^2+^ induces a K^+^ efflux followed by Cl^−^ and water resulting in cell dehydration. Of note, human RBCs are unable to regulate their volume (9, 10), meaning once KCl and water are lost, the RBC will remain dehydrated, which impairs the rheological properties of RBC (11, 12). Hence, the activity of KCNN4 must be tightly controlled in RBC to prevent rheological issues. Whereas the physiological role of KCNN4 in RBC is still largely ignored, it is involved in two major RBC diseases: sickle cell anemia and malaria. In sickle cell anemia, KCNN4 activation induces catastrophic and irreversible dehydration causing the precipitation of hemoglobin within the cells (13), and in malaria, a degree of dehydration makes it difficult for the malarial parasite to multiply within the RBC, limiting its cycle (14). In addition, we and others have shown that gain-of-function mutations in KCNN4 lead to RBC fragility and hemolytic anemia (15, 16, 17), hallmark of the rare disease dehydrated hereditary stomatocytosis also named hereditary xerocytosis (18, 19, 20, 21). Up to now, this is the only link between a pathology and gain-of-function mutations in *KCNN4*. However, KCNN4 has been involved in other pathologies. It was shown to promote cell proliferation and migration, and it is investigated as an oncogene in human cancers, particularly in glioblastoma (22) (for review, see Refs. (23, 24, 25)). Moreover, its activity in microglia suggests its involvement in different neurological disorders, such as Alzheimer disease, ischemic stroke, and multiple sclerosis (25, 26). This channel is thus considered as a promising candidate to treat different human conditions: RBC pathologies but also cancer, cardiac arrhythmia, as well as neurological disorders (25, 27, 28, 29, 30, 31).

The present study was designed to better understand the regulation of KCNN4 activity in RBC, as its activation might be deleterious for volume homeostasis and rheological properties. We investigated the Ca^2+^ dependency of KCNN4 in RBC and observed a discrepancy between the level of intracellular Ca^2+^ increase and KCNN4-induced dehydration. Using human embryonic kidney 293 (HEK293) or K562-transfected cells, we showed that KCNN4 activity was inhibited by the Ca^2+^ ATPase, PMCA4b (plasma membrane calcium-transporting ATPase 4b), through a molecular interaction between the channel and the C-terminal end of the pump. This control by the Ca^2+^ pump was not restricted to KCNN4 but also observed with another member of the Ca^2+^-dependent K^+^ channel family, KCNN2. This is the first characterization of the control of Ca^2+^-dependent K^+^ channels by molecular interactions with a Ca^2+^ pump. This mechanism could be a new target to treat disorders that are linked to KCNN4 activity.

## Results

In RBC, we compared dynamic changes in intracellular water, K^+^, and Na^+^ contents under different experimental conditions designed to increase intracellular Ca^2+^ concentration in order to activate KCNN4. Yoda1 was used to stimulate PIEZO1 (32, 33), the nonselective cation conductance mechanically gated (34). In RBC, PIEZO1 stimulation induces a nonspecific cation flux, allowing K^+^ loss and Na^+^ and Ca^2+^ uptake (35, 36). This Ca^2+^ entry leads to KCNN4 activation allowing K^+^ efflux followed by Cl^-^ and H_2_O resulting in RBC dehydration. Vanadate was used to inhibit the Ca^2+^ ATPase pump, which is very active in RBC, maintaining a cytoplasmic Ca^2+^ concentration of around 100 nM, against extracellular calcium concentration in the millimolar range (37, 38). Finally, we used the Ca^2+^ ionophore 4Br-A23187. In RBC, which have no intracellular Ca^2+^ stores, 4Br-A23187 is expected to equilibrate calcium across the plasma membrane according to its electrochemical gradient, overwhelming the activity of the calcium pump. Ouabain was present in all studies to prevent movement of Na^+^ and K^+^ *via* the Na^+^–K^+^ pump.

**Figure 1:**
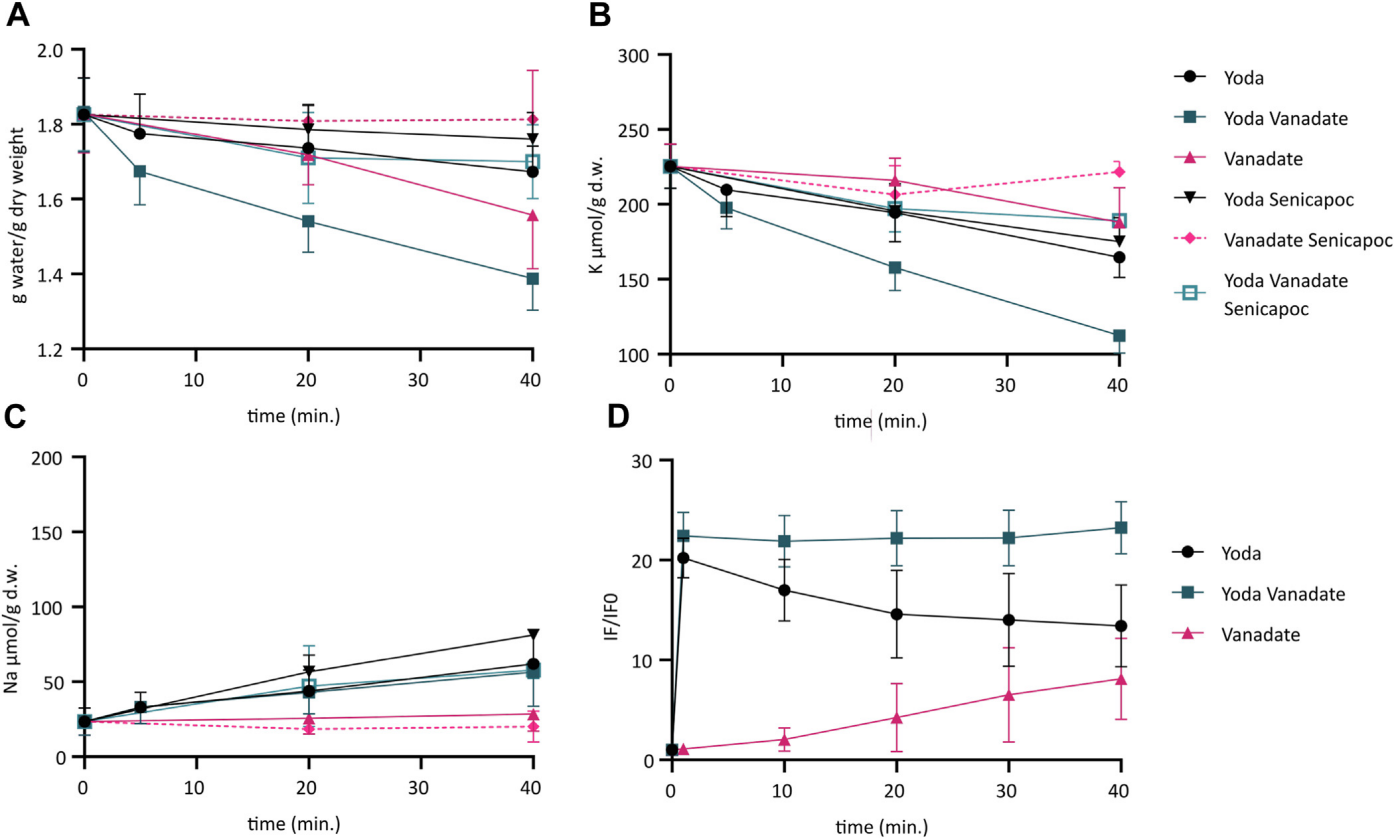
**Water, Na**^**+**^**, K**^**+**^**, and Ca**^**2+**^**contents of RBC in different experimental conditions.** Time course of water (*A*), K^+^ (*B*), and Na^+^ (*C*) contents was measured on washed RBC treated with 2 μM Yoda1 ± 5 mM vanadate or 5 mM vanadate alone (similar results were obtained with 1 mM vanadate, Fig. S1). Senicapoc (4 μM) was added to block KCNN4. Data are means ± SD with only one side shown for clarity purposes. Statistical analyses were done with two-way ANOVA and Tukey correction. For water measurements, comparison *t* = 0 *versus* Yoda1 condition: *p* = 0.574 for *t* = 5’; *p* = 0.046 for *t* = 20’; *p* = 0.017 for *t* = 40’; comparison *t* = 0 *versus* Yoda1 + vanadate: *p* ≤ 0.0001 at all time points; comparison *t* = 0 *versus* vanadate: *p* = 0.009 for *t* = 20’; *p* < 0.0001 for *t* = 40’. For K^+^ measurements, comparison *t* = 0 *versus* Yoda1 condition: *p* = 0.022 for *t* = 5’; *p* < 0.0001 for *t* = 20′ and *t* = 40’; comparison *t* = 0 *versus* Yoda1 + vanadate: *p* < 0.0001 at all time points; comparison *t* = 0 *versus* vanadate: *p* = 0.617 for *t* = 20’; *p* < 0.0001 for *t* = 40’. For Na^+^ measurements: comparison *t* = 0 *versus* Yoda1: *p* = 0.748 for *t* = 5′, *p* = 0.0003 for *t* = 20′, *p* = 0.0014 for *t* = 40’; comparison *t* = 0 *versus* Yoda1 + vanadate: *p* = 0.812 for *t* = 5′, *p* = 0.0045 for *t* = 20′, and *p* = 0.0005 for *t* = 40’; comparison *t* = 0 *versus* vanadate: *p* > 0.9999 for *t* = 20′ and *t* = 40’. *D,* fluorescence associated with variations in intracellular Ca^2+^ contents measured by flow cytometry with Fluo4-AM probe. Ca^2+^-associated fluorescence was measured overtime after addition of 2 μM Yoda1 ± vanadate (5 mM) or vanadate (5 mM) alone. Similar results were obtained with 1 mM vanadate. Data are expressed as a ratio of mean fluorescence at different time over mean fluorescence at *t* = 0. A two-way ANOVA mixed model with Greenhouse–Geisser and Tukey corrections was done. *p* Values were <0.0001 when comparing the different conditions for a single time except *t* = 10′ Yoda1 *versus* Yoda1 + vanadate *p* = 0.0025 and *t* = 40′ Yoda1 *versus* vanadate *p* = 0.0002. RBC, red blood cell.

Fig. 1 shows changes in water, Na^+^, K^+^, and Ca^2+^ contents in RBC. RBC water contents were correlated with Na^+^ and K^+^ net fluxes, and resulting total Na^+^ + K^+^ contents are shown in Table S1. Vanadate alone (*pink triangles*) induced a K^+^ loss (with water loss) without significant change in intracellular Na^+^ content. With Yoda1 (*black circles*), the K^+^ loss was greater than the Na^+^ uptake. This effect was more prominent in the additional presence of vanadate (*blue squares*). That the excess K^+^ loss was due to KCNN4 activation was shown by the effect of KCNN4 blocker Senicapoc (*open blue squares*). In the presence of Senicapoc, there was no significant difference between the net Na^+^ uptake and the net K^+^ loss at any time in any condition (Table S1), consistent with the idea that excess K^+^ loss (over Na^+^ gain) is always mediated by KCNN4. K^+^ fluxes were not affected by the addition of 0.1 mM bumetanide, used to inhibit NKCC and KCC transporters (Table S2). The present data are consistent with the idea that PIEZO1 is driving a Na^+^ uptake compensated by a K^+^ efflux, with KCNN4 mediating an excess K^+^ loss leading to RBC dehydration (39), confirming our previous data (35).

Fig. 1*D* illustrates the mean fluorescence (as Fluo4 fluorescence) associated with changes in intracellular Ca^2+^ concentration. The Ca^2+^ fluorescence dramatically increased in RBC immediately after addition of Yoda1 to the RBC suspension (*black circles*), the fluorescence then fell by about 40% over 40 min. In the presence of Yoda1 plus vanadate (*blue squares*), there was a sustained elevation of intracellular Ca^2+^. Vanadate alone induced a slow increase of Ca^2+^ fluorescence, no doubt because of inward calcium leaks (40). Despite a large increase in Ca^2+^ fluorescence with Yoda1, the KCNN4-mediated K^+^ loss (*i.e.*, the Senicapoc-sensitive K^+^ loss, Fig. 1*B*) was small. By contrast, a relatively lower change in Ca^2+^ level with vanadate alone activated KCNN4 more efficiently.

**Figure 2:**
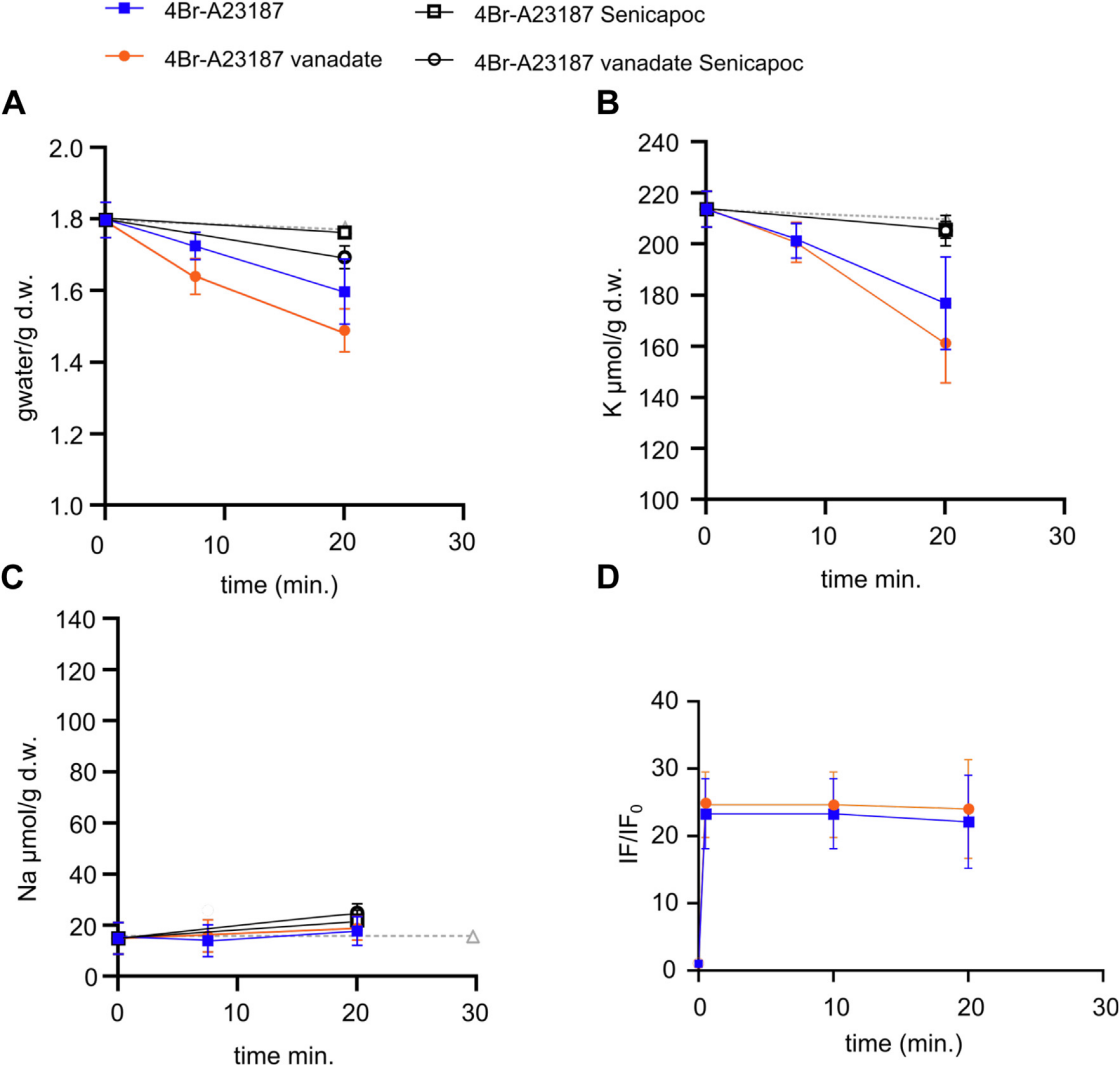
**Effect of 4Br-A23187 and vanadate on water and ion permeability of RBC.** Effect of 4Br-A23187 (5 μM) (*blue*) and 4Br-A23187 + vanadate (5 mM) (*orange*) on water (*A*), K^+^ (*B*), and Na^+^ (*C*) contents and Ca^2+^ fluorescence (*D*): ratio of Fluo-4-associated fluorescence at different times over fluorescence at *t* = 0 (IF_0_ = 468 and 750 AU for 4Br-A23187 and 4Br-123187 + vanadate, respectively). Control measurements were given with *gray dash line* (*triangle*) for comparison. The effect of Senicapoc (4 μM), inhibitor of KCNN4, was assessed on water, Na^+^, and K^+^ contents, in the presence of 4Br-A23187 (5 μM) (*empty squares*) and 4Br-A23187 + vanadate (5 mM) (*empty circles*). Means ± SD of 12 independent samples from four experiments (4Br-A23187 and 4Br-A23187 + vanadate), six independent samples from two experiments (Senicapoc conditions), one sample from four experiments for Ca^2+^. For water contents: comparison *t* = 0 *versus t* = 5 min: 4Br-A23187: *p* = 0.033; 4Br-A23187 + vanadate: *p* < 0.0001; comparison *t* = 0 *versus t* = 20 min 4Br-A23187: *p* < 0.0001; 4Br-A23187 + vanadate: *p* < 0.0001; 4Br-A23187 + Senicapoc: *p* = 0.5913 and 4Br-A23187 + vanadate + Senicapoc: *p* = 0.0010. Comparison of 4Br-A23187 *versus* A23187 + vanadate at *t* = 5 min *p* = 0.0069, at *t* = 20 min *p* = 0.0003; at *t* = 20 min comparison of 4Br-A23187 *versus* A23187 + Senicapoc *p* < 0.0001; A23187 + vanadate *versus* A23187 + vanadate + Senicapoc: *p* < 0.0001. For K^+^ contents, comparison *t* = 0 *versus t* = 5 min: 4Br-A23187: *p* = 0.0743; 4Br-A23187 + vanadate: *p* = 0.0505; comparison *t* = 0 *versus t* = 20 min 4Br-A23187: *p* < 0.0001; 4Br-A23187 + vanadate: *p* < 0.0001; 4Br-A23187 + Senicapoc: *p* = 0.5371 and 4Br-A23187 + vanadate + Senicapoc: *p* = 0.3809. Comparison of 4Br-A23187 *versus* A23187 + vanadate at *t* = 5 min: *p* > 0.999, at *t* = 20 min: *p* = 0.0097. At *t* = 20 min comparison of 4Br-A23187 *versus* A23187 + Senicapoc *p* = 0.0001; A23187 + vanadate *versus* A23187 + vanadate + Senicapoc: *p* < 0.0001. For Na^+^ contents, comparison *t* = 0 *versus t* = 5 min: 4Br-A23187: *p* = 0.9807; 4Br-A23187 + vanadate: *p* = 0.9930; comparison *t* = 0 *versus t* = 20 min 4Br-A23187: *p* = 0.3992; 4Br-A23187 + vanadate: *p* = 0.0061; 4Br-A23187 + Senicapoc: *p* = 0.2367 and 4Br-A23187 + vanadate + Senicapoc: *p* = 0.0002. Comparison of 4Br-A23187 *versus* A23187 + vanadate at *t* = 5 min: *p* > 0.999, at *t* = 20 min: *p* = 0.0097. At *t* = 20 min comparison of 4Br-A23187 *versus* A23187 + Senicapoc *p* = 0.9984; A23187 + vanadate *versus* A23187 + vanadate + Senicapoc: *p* = 0.6737. RBC, red blood cell.

To further assess the effect of vanadate on KCNN4 activity, we used the Ca^2+^ ionophore 4Br-A23187 (Fig. 2). Following ionophore addition, the intracellular Ca^2+^ reached a plateau within seconds (Fig. 2*D*, *filled orange circles*). The Ca^2+^ fluorescence of RBC treated by 4Br-A23187 alone or together with vanadate (panel *D*, *filled blue squares*) plateaued at a similar level, suggesting a similar intracellular calcium concentration under both conditions. Despite this similarity, the K^+^ loss and RBC dehydration were larger in the presence of vanadate (Fig. 2, *A* and *B*). Both were blocked by addition of Senicapoc, confirming the involvement of KCNN4 in the K^+^ and water loss. Hence, the increased activity of KCNN4 observed with addition of vanadate (panels *A* and *B*) was apparently not attributable to any difference in internal calcium but seemed to be secondary to the presence of vanadate itself.

These data suggested that the Ca^2+^ pump activity could impair KCNN4 functioning. We reconstituted the system in HEK293T cells cotransfected with PIEZO1 and KCNN4 with or without PMCA4b (the major form of PMCA in RBC) (41, 42).

**Figure 3:**
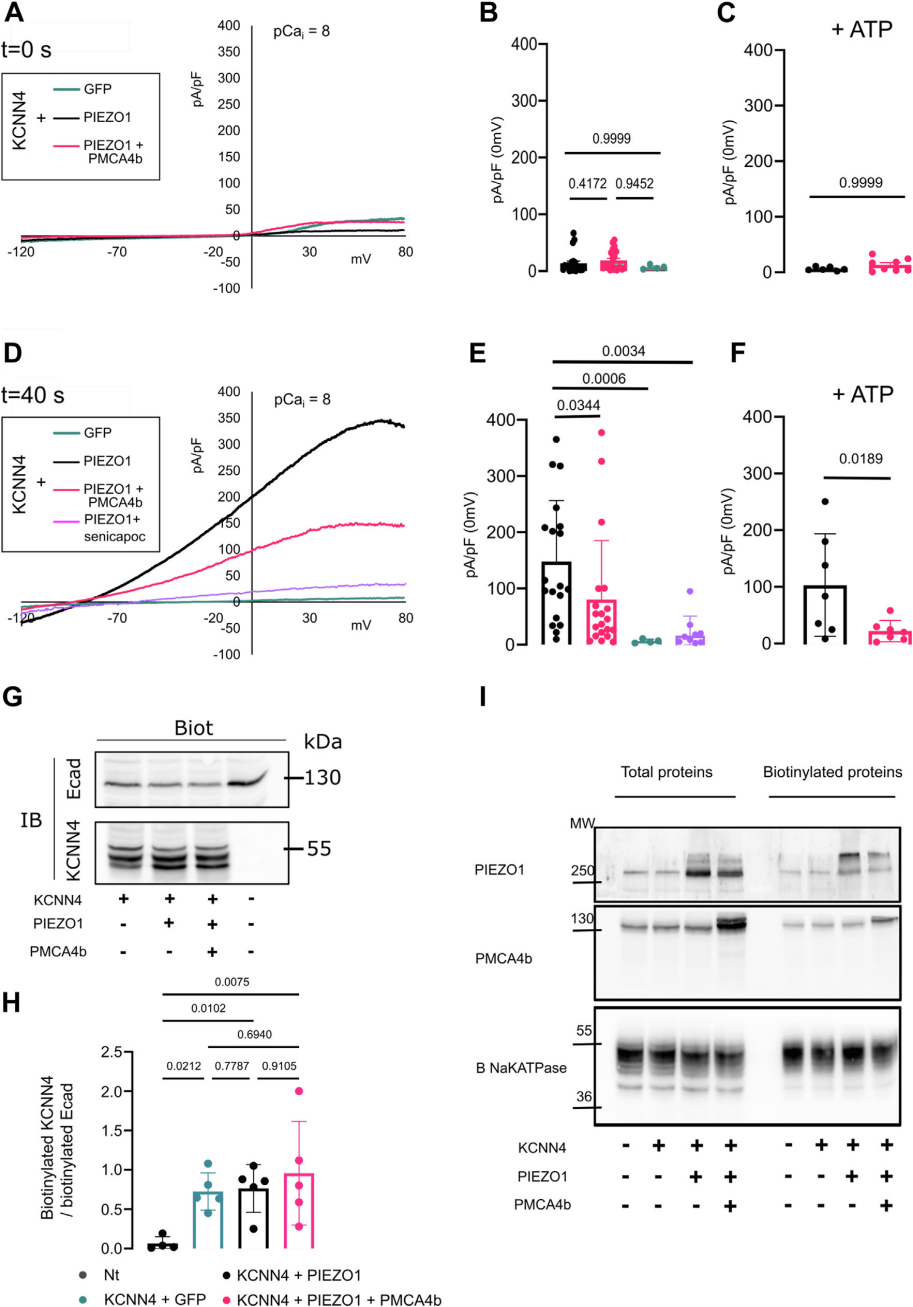
**Electrophysiological recordings of PIEZO1-mediated KCNN4 current on HEK293T cells transfected with KCNN4, PIEZO1, and PMCA4b.***A* and *D,* representative traces in different cotransfection conditions: KCNN4 + GFP (*green*), KCNN4 + PIEZO1 (*black*), and KCNN4 + PMCA4b (*pink*) in whole-cell configuration at *t* = 0 (*A*) and *t* = 40 s (*D*) after 15 μM Yoda1 addition. *Purple trace* illustrated the effect of 300 nM Senicapoc, blocker of KCNN4, on the PIEZO1-induced current in HEK293T cells cotransfected with PIEZO1 + KCNN4. Associated current density (pA/pF) at 0 mV measured at *t* = 0 (*B*) or *t* = 40 s (*E*) after Yoda1 addition without ATP inside or with 1 mM ATP at *t* = 0 (*C*) and at *t* = 40 s (*F*). *n* = 4 (KCNN4 + GFP) to *n* = 18 (KCNN4 + PIEZO1 + PMCA4b) cells coming from different batches of transfection. Data are means ± SD. *p* Values are written on the figures. *G* and *H,* expression of KCNN4 at the plasma membrane when PIEZO1 or PIEZO1 + PMCA4b were cotransfected. *G,* representative Western blot and (*H*) quantitative analysis of five different Western blots done on three different transfection assays showing proteins addressed at the plasma membrane, only biotinylated proteins were detected. KCNN4 is glycosylated and appeared as multiple bands around 50 kDa. The bands were more or less discrete depending on experiments. KCNN4 expression level was normalized to the level of biotinylated E-cadherin. Data are means ± SD, n = 5. *I,* immunodetection of PIEZO1 and PMCA in HEK293T cells: detection of total proteins and biotinylated proteins indicating plasma membrane expression. Endogenous PMCA was detected by the anti-PMCA antibody; the transfected PMCA tagged with GFP was a bit above endogenous PMCA. Immunodetection of the Na-K-ATPase ß subunit was used as loading reference. HEK293T, human embryonic kidney 293T cell line; PMCA4b, plasma membrane calcium-transporting ATPase 4b.

**Figure 4:**
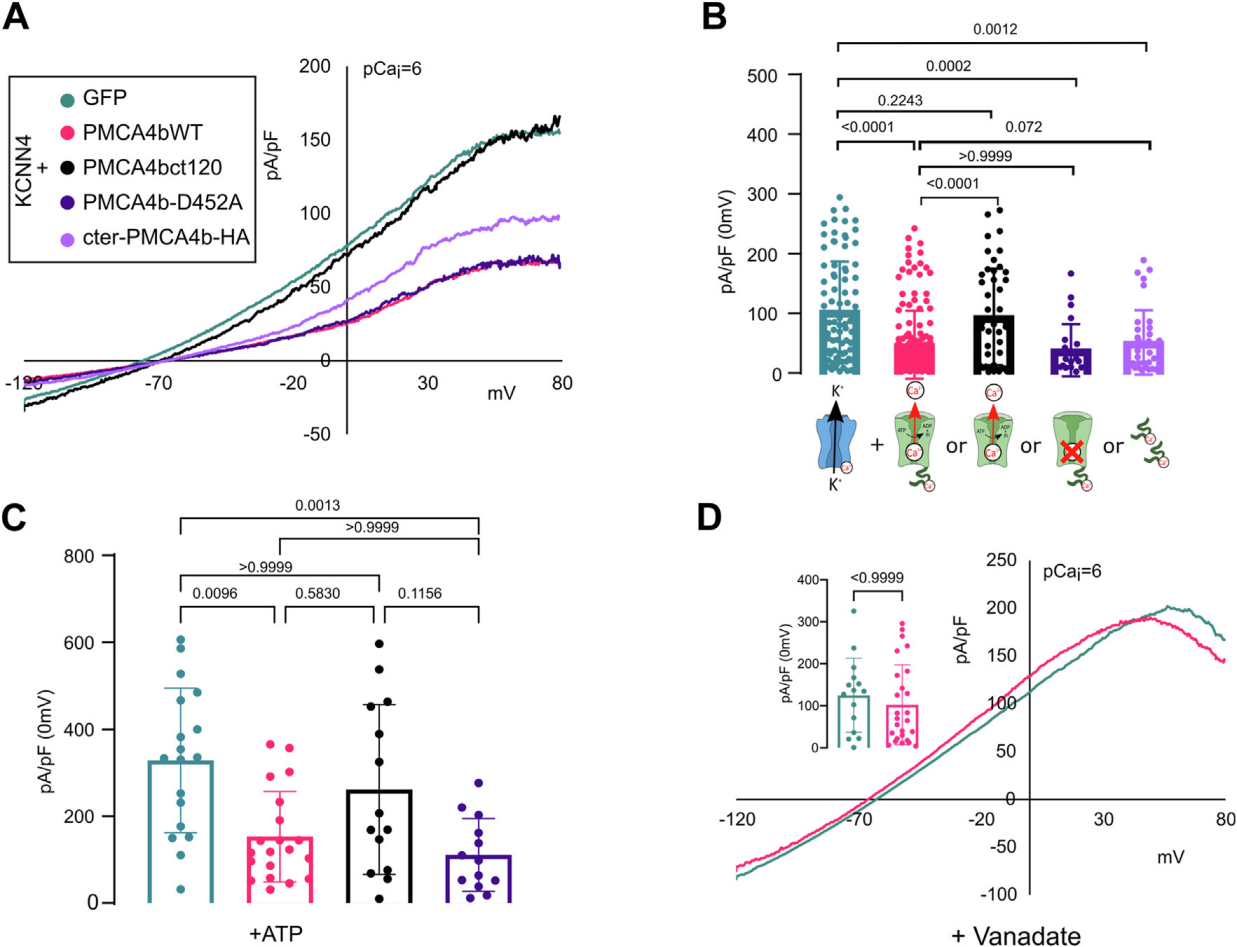
**Electrophysiology on HEK293T cells**. *A*–*D,* HEK293T cells expressed KCNN4 with or without PMCA4b WT or mutated in the presence of 1 μM Ca^2+^ in the pipette (pCai = 6). *A,* representative current traces observed in HEK293T cells expressing KCNN4 with GFP (*green*) or with different PMCA4b constructs: WT (*pink*), deleted of the C-terminal end (PMCA4bCT120, *black*), mutated D452A (*sapphire*), or soluble PMCA4b C-terminal end (*purple*). *B,* corresponding current density in pA/pF measured at 0 mV, n = 111 KCNN4 + GFP, n = 133 KCNN4 + PMCA4b WT, n = 73 KCNN4 + PMCA4bCT120, n = 24 KCNN4 + PMCA4b D452A and n = 39 KCNN4 + soluble PMCA4b C-terminal end. *C,* current density measured in the presence of 1 mM ATP for HEK293T cells expressing KCNN4 + GFP (*green*), KCNN4 + PMA4b WT (*pink*), KCNN4 + PMCA4bCT120 (*black*), and KCNN4 + mutated D452A (*sapphire*). *D,* representative traces and current density in pA/pF in the presence of 5 mM vanadate incubated for 20 min in extracellular bath. Data are means ± SD. *p* Values are indicated above bars. HEK293T, human embryonic kidney 293T cell line; PMCA4b, plasma membrane calcium-transporting ATPase 4b.

Without PIEZO1 stimulation (10 nM intracellular Ca^2+^), no KCNN4 current was observed (Fig. 3, *A* and *B*). Within 40 s following PIEZO1 stimulation by Yoda1, a Senicapoc-sensitive current was recorded corresponding to KCNN4 activity (Fig. 3, *D* and *E*). The activation of KCNN4 was reduced upon coexpression of PMCA4b (Fig. 3, *D* and *E*). This decreased response was not related to an impaired surface expression of KCNN4 (Fig. 3, *G* and *H*). Fig. 3*I* showed that PIEZO1 and PMCA4b were correctly expressed at the plasma membrane. We challenged whether the reduction of the current could result from a faster decrease in Ca^2+^ concentration because of PMCA4b activity. Intracellular Ca^2+^ was clamped at 1 μM. Despite a constant calcium concentration, KCNN4 current was again reduced in the presence of PMCA4b (Fig. 4, *A* and *B*, *pink versus emerald traces*). The inhibition of KCNN4 activity by PMCA4b expression could also result from local Ca^2+^ pumping out around KCNN4. To assess this hypothesis, we used two different constructs of PMCA4b: the PMCA4b-ct120 deleted of 120 C-terminal amino acids and the point mutated PMCA4b-D452A. PMCA4b-ct120 is constitutively active (43), whereas the D452A mutation prevents a phosphorylation on D452, which inactivates the Ca^2+^ pump (44). Fig. 4, *A* and *B* shows that the constitutively active PMCA4b-ct120 was no longer able to inhibit KCNN4 activity (*black trace*) in contrast to the inactive PMCA4b-D452A (*dark blue trace*). The inhibitory effect of PMCA4b on KCNN4 was not dependent on the activity of the Ca^2+^pump. To confirm this hypothesis, we repeated these experiments in the presence of ATP ensuring energy supply throughout recording (Fig. 3, *C*–*F* and 4*C*). The difference between control and PMCA4b condition was equivalent in the presence or the absence of ATP. This ruled out the role of the Ca^2+^ pump activity on KCNN4 inhibition. We then assessed the effect of vanadate on KCNN4 current in HEK293T cells expressing KCNN4 alone (Fig. 4*D*, *turquoise trace*) or KCNN4 + PMCA4b (Fig. 4*D*, *pink trace*). With vanadate, the current mediated by KCNN4 was similar in the absence or the presence of PMCA4b, suggesting that vanadate was able to prevent the inhibition of KCNN4 current by PMCA4b. As vanadate is not a specific inhibitor of the calcium pump, we checked that vanadate by itself did not alter KCNN4 activity (Fig. S2). To resume, the channel was inhibited when a mutation inactivated the Ca^2+^ pump, whereas it was not inhibited when the Ca^2+^ pump was blocked by vanadate. This suggested that the structure of the pump could play a role in the inhibition of the channel, rather than its Ca^2+^ pumping out activity. According to the results with PMCA4b-ct120 (pump deleted of its C-terminal end), we hypothesized that the C-terminal part of the pump was responsible for the reduced activity of KCNN4. We expressed the C-terminal end of PMCA4b (cter-PMCA4b-HA) in HEK293T cells with KCNN4. Fig. 4, *A* and *B* (*purple trace*) shows that the expression of this cytoplasmic domain of the pump reduced KCNN4 current.

**Figure 5:**
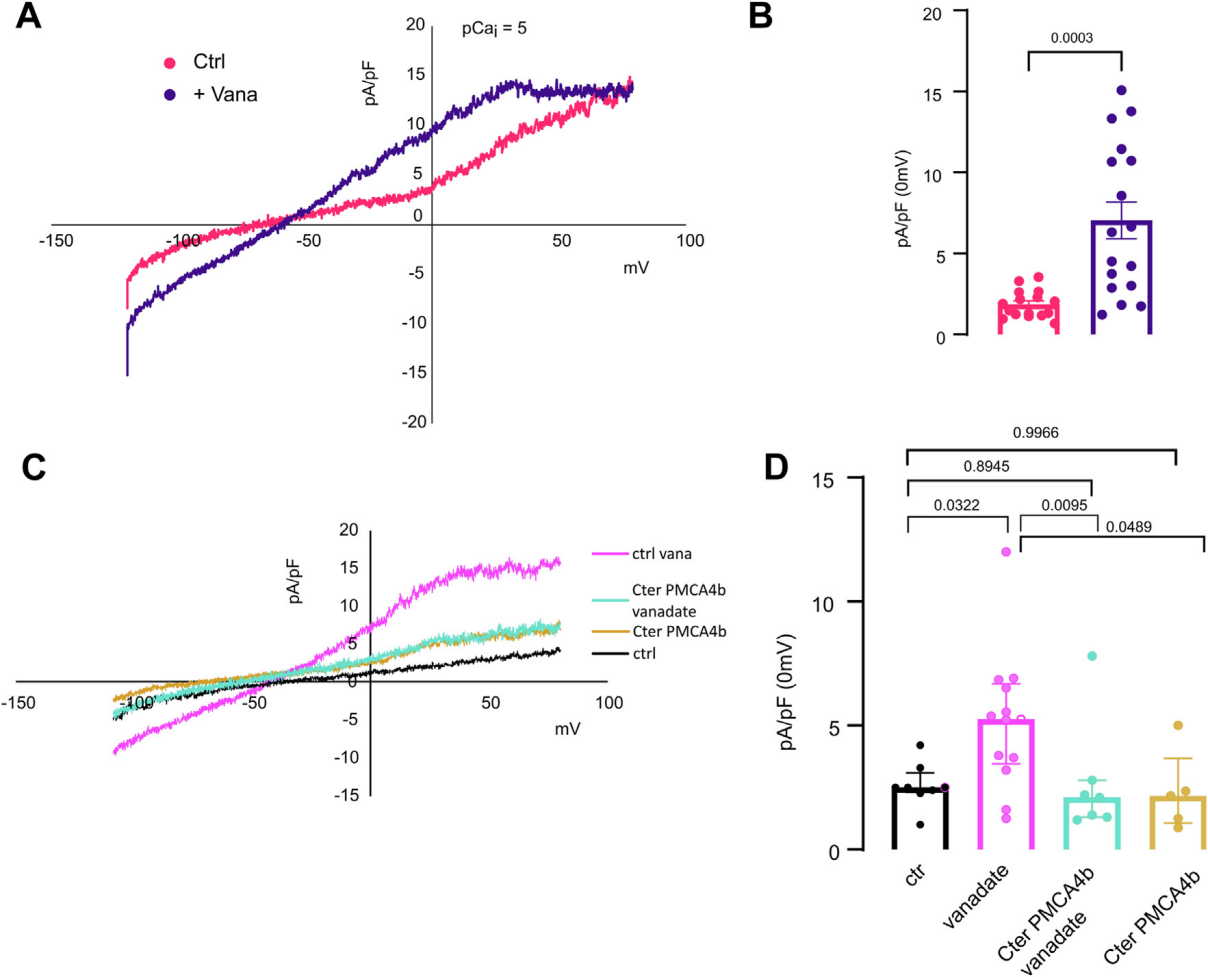
**Endogenous KCNN4 currents in K562 cells**. *A,* representative traces of endogenous current with (*purple*) or without (*magenta*) 5 mM vanadate in the pipette, measured with patch-clamp in whole-cell configuration with 10 μM Ca^2+^-free inside. *B,* corresponding statistical analyses, means ± SD. n = 16 for control and n = 17 for vanadate. *C,* representative current traces observed in K562 cells: control (transfected with pIRES-eGFP) ± 50 μM vanadate or expressing Cter PMCA4b ± 50 μM vanadate. pCa_i_ = 5. *D,* current density (pA/pF) in control K562 cells (transfected with pIRES-eGFP) (*black dots*) in the presence of 50 μM vanadate (*pink dots*) or in K562 cells expressing Cter PMCA4b-HA with 50 μM vanadate (*green*) or without vanadate (*yellow*). Data are medians ± interquartile range (5–13 cells coming from three batches of transfection). PMCA4b, plasma membrane calcium-transporting ATPase 4b.

In an attempt to assess the role of the C-terminal part of PMCA4b on endogenous KCNN4 currents, we reproduced these experiments on native currents in erythroleukemia K562 cell line, which endogenously expressed KCNN4 and PMCA4b (Fig. 5*A* and Fig. S3 showing current inhibition by Senicapoc) (45). The endogenous Ca^2+^-dependent K^+^ current was increased when vanadate (5 mM as well as 50 μM) was added in the pipette with 10 μM free Ca^2+^ (Fig. 5*B*). Moreover, in the presence of CterPMCA4b-HA, the activating effect of vanadate on KCNN4 endogenous current was blocked (Fig. 5, *C* and *D*). This confirms that a direct effect of vanadate on KCNN4 activity is unlikely. Hence, KCNN4 inhibition by PMCA4b appeared related to the conformation of the pump: it is abolished when vanadate binds to the pump, and it is mediated by its C-terminal part.

**Figure 6:**
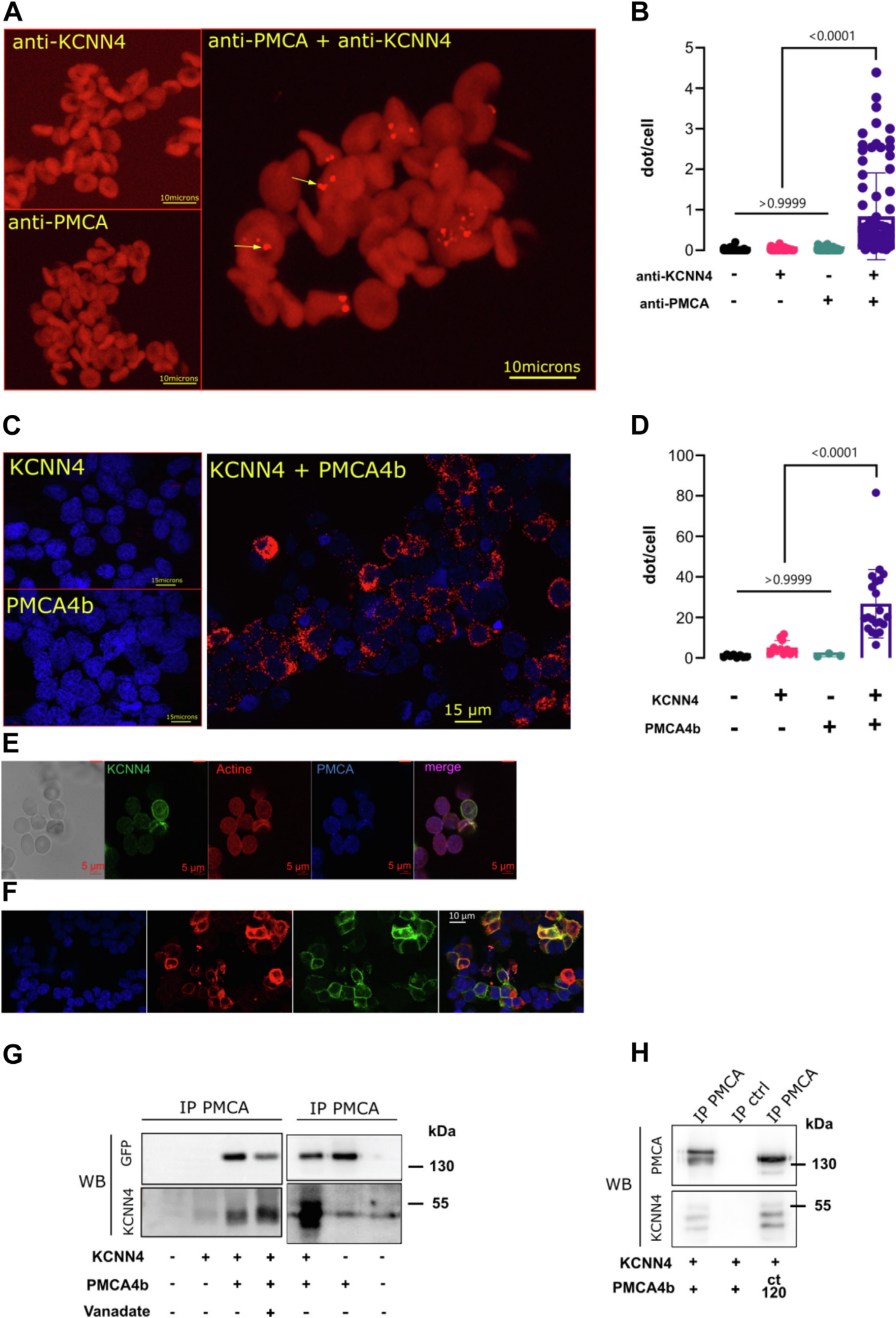
**Proximity ligation assay (PLA) and immunoprecipitation.** Representative pictures of PLA on RBC (*A*) or on HEK293T cells coexpressing KCNN4 and PMC4Ab (*B*) done with both antibodies anti-KCNN4 and anti-PMCA or with only anti-PMCA or anti-KCNN4. The fluorescence covering the RBC is due to hemoglobin (Hb) fluorescence. The high Hb concentration together with fixation allowed Hb to emit at 624 nm wavelength used to detect PLA signal. Quantification of PLA dots per cell in different experimental conditions for RBC (*C*) and HEK293T cells expressing KCNN4 and/or PMCA4b (*D*). Data are means ± SD, n = 2 experiments for HEK293T-transfected cells and n = 4 for RBC. *E,* immunofluorescence on RBC fixed in 1X PBS containing 4% paraformaldehyde and 0.05% glutaraldehyde and blocked in 1X PBS, 4% bovine serum albumin, and 1% goat serum. Cells were labeled with antibodies used for PLA experiments in the same conditions: anti-KCNN4 (1/200 dilution, rabbit against peptide 28–427; Proteintech) or anti-PMCA (1/200 dilution, mouse; Invitrogen) and with Alexa 568 Phalloidin (1/200 dilution). Secondary antibodies (1/5000 dilution) anti-rabbit Alexa 588 or anti-mouse Alexa 647 (From l*eft* to *right*: transmission, KCNN4 immunodetection, actin detection with phalloidin, PMCA detection, merged KCNN4–actin–PMCA images). There was no signal with secondary antibodies used alone. Images captured with confocal microscope Zeiss LSM880. *F,* immunofluorescence on HEK293T cells transfected with KCNN4 and PMCA4b WT. Cells were fixed in the same conditions as for PLA experiments and blocked in 1X PBS, 4% bovine serum albumin, and 1% goat serum. Cells were labeled with anti-KCNN4 (1/200 dilution, rabbit against peptide 28–427; Proteintech) and secondary antibodies (1/5000 dilution) anti-rabbit Alexa 647. PMCA4B was detected with tagged GFP (488). From *left* to *right*: nucleus labeling with DAPI (*blue*), KCNN4 immunodetection (*red*), PMCA (*green*), merged images. There was no signal with secondary antibodies used alone. Images captured with confocal microscope Zeiss LSM880. *G,* immunoprecipitation of PMCA4b with anti-PMCA (mab 5F10; Invitrogen): HEK293T cells transfected with KCNN4 or KCNN4 + PMCA4b WT or PMCA4b WT or not transfected (control). Eluted fractions were revealed on WB with anti-KCNN4 (rabbit IgG; Proteintech) and anti-GFP (rabbit IgG; Cell Signaling) to detect PMCA4b that is fused to GFP. *H,* immunoprecipitation of PMCA4b with anti-PMCA (mab 5F10; Invitrogen): HEK293T cells transfected with PMCA4b WT or KCNN4 + PMCA4b WT or KCNN4 + MCA4bCT120. Control immunoprecipitation (IP ctrl) was done on HEK293T cells transfected with KCNN4 + PMCA4b WT loaded on empty agarose beads. Eluted fractions were revealed on WB with anti-PMCA (mab 5F10) and anti-KCNN4 (rabbit IgG; Proteintech). Representative WB, n = 4. DAPI, 4′,6-diamidino-2-phenylindole; HEK293T, human embryonic kidney 293T cell line; PMCA4b, plasma membrane calcium-transporting ATPase 4b; RBC, red blood cell.

To address a possible molecular interaction, a proximity ligation assay (PLA) was done on RBC and HEK293T cells expressing KCNN4 and different PMCA4b constructs. Fig. 6, A–D showed a proximity between KCNN4 and PMC4Ab detected in HEK293T cells and confirmed in RBC. Of note, few dots were observed in RBC. The antibodies were efficient in immunofluorescent assay suggesting accessibility of the epitopes (Fig. 6, *E* and *F*). It should be noticed that the number of KCNN4 proteins in RBC plasma membrane has been debated for many years. Depending on the methods used to estimate the number of KCNN4 channel per RBC, it varies between ≈1 to 5 and ≈3000 (46, 47, 48, 49, 50). The very small number of KCNN4–PMCA4b interaction detected in RBC could indicate either a small number of channels or, not all the channels and Ca^2+^ pumps were in close proximity. Alternatively, only a faint proportion of these complexes was detectable because of impaired DNA polymerization caused by the highly glycosylated RBC membrane. The greater PLA signal in HEK293T could indicate a more efficient reaction in these cells as well as more interacting proteins. To confirm these results, a coimmunoprecipitation (co-IP) was done on HEK293T cells expressing KCNN4 with PMCA4b or the truncated pump PMCA4b-ct120. PMCA4b IP was able to specifically drag KCNN4 in HEK293T-transfected cells (Fig. 6, *G* and *H*). Interestingly, PMCA4b-ct120 was still interacting with KCNN4 as shown by co-IP (Fig. 6*H*).

**Figure 7:**
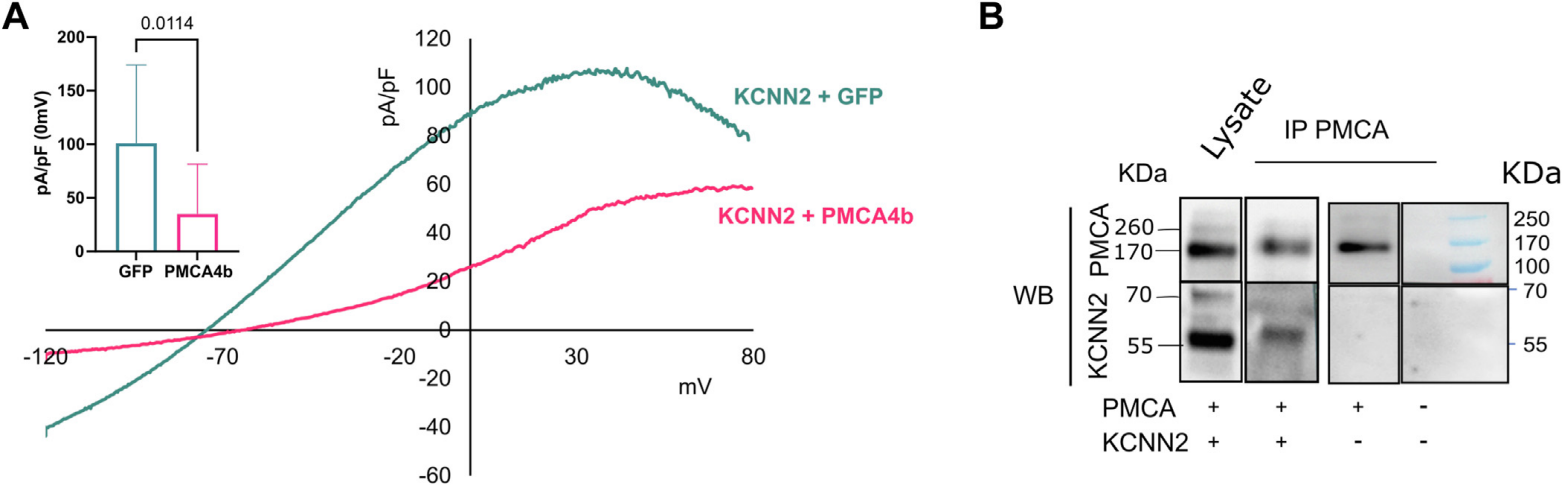
**Control of KCNN2 by PMCA4b in HEK293T cells.***A,* representative traces of currents in HEK293T cells expressing KCNN2 in the presence (*magenta*) or the absence (*indigo*) of PMCA4b and statistical analysis in *inset* (bar plot with mean ± SD, n = 7 for KCNN2 + GFP, n = 11 for KCNN2 + PMCA4b, *p* value calculated with a Mann–Whitney test). *B,* immunoprecipitation with anti-PMCA (mab 5F10; Invitrogen) of HEK293T cells transfected with KCNN2 + PMCA4bWT or PMCA4bWT or not transfected. Lanes coming from two different blots have been reorganized on the figure. Western blots were revealed with anti-PMCA (mab 5F10) and anti-KCNN2 antibodies (rabbit against rat KCNN2). HEK293T, human embryonic kidney 293T cell line; PMCA4b, plasma membrane calcium-transporting ATPase 4b.

We then wondered whether other members of the Ca^2+^-dependent K^+^ channel family could also be regulated by PMCA4b. We transfected KCNN2, a small conductance member of the family, in HEK293T cells, with or without PMCA4b. The current in HEK293T cells transfected with KCNN2 was sensitive to Dab7, specific inhibitor of this channel (Fig. S4). As for KCNN4, KCNN2 current was reduced in the presence of PMCA4b (Fig. 7).

## Discussion

The experiments in RBC suggested a control of KCNN4 activity by the Ca^2+^ pump that exceeds the effect of intracellular Ca^2+^ increase. Vanadate enhanced the activation of KCNN4 induced by either the stimulation of PIEZO1 or the calcium ionophore 4Br-A23187 despite intracellular Ca^2+^ being sufficient to drive optimal activation of KCNN4 in both experimental conditions. Vanadate is a nonspecific inhibitor of ATPases, and it targets tyrosine kinases and phosphatases as well as P-type ATPases. To overcome the absence of vanadate specificity, we used HEK293T cells that provided evidence for a direct inhibitory interaction of the PMCA4b on KCNN4 that was not dependent on Ca^2+^ pumping-out activity. The inhibition involved the C-terminal soluble part of PMCA4b that was necessary and sufficient to recapitulate the effect of PMCA4b overexpression. Its deletion from the protein abolished the inhibition of KCNN4, whereas its expression as a soluble part reduced the activity of KCNN4 in HEK293T-transfected cells as in K562 cells (endogenous currents). We propose that PMCA4b is part of a molecular mechanism controlling KCNN4 activity.

The molecular mechanism involved in the inhibition of KCNN4 by PMCA4b might be envisioned as follows. PMCA4b and KCNN4 form a molecular complex that is not dependent on the C-terminal end of PMCA4b (truncated PMCA4B coimmunoprecipitated with KCNN4). Moreover, this interaction is not linked to the functional state of PMCA4b: the co-IP between PMCA4b and KCNN4 was observed in the presence or the absence of vanadate. Vanadate blocks the Ca^2+^ pump in the E2P state, phosphorylated unable to transport Ca^2+^ (51, 52, 53). This E2P state was able to interact with KCNN4. However, this conformation removed the molecular control of the PMCA4b C-terminal end on KCNN4 activity. Hence, in the PMCA4b–KCNN4 complex, the C-terminal end of PMCA4b could modulate KCNN4 activity in a manner depending on PMCA4b conformation. To explain the effect of vanadate on KCNN4 in K562 cells or RBC, we propose that vanadate, by altering PMCA4b conformation, removed a tonic inhibition mediated by the C-terminal end of PMCA4b on KCNN4 conductance. This suggests that KCNN4 activity is constitutively repressed in the KCNN4–PMCA4b complex, and the inhibition disappears upon changes in the conformation of PMCA4b. Two distinct parts of PMCA4b are thus involved in an interaction with KCNN4: one that links the two proteins and another one that reduces KCNN4 activity.

The structure of PMCA4b has not yet been solved; its spatial organization can be modelized according to the structure of the P-type ATPase SERCA (52). Five structural domains are proposed: a membrane spanning domain with 10 α helices and 4 cytoplasmic domains: actuator, phosphorylation, nucleotide binding, and regulatory domains. The regulatory domain is the C-terminal part of the protein that has two calmodulin (CaM)-binding sites (44, 54). CaM adjusts pump activity to Ca^2+^ concentration (41, 55). In low Ca^2+^, PMCA4b is autoinhibited by its C-terminal end, which blocks catalytic domain. Upon Ca^2+^ increase, CaM–Ca^2+^ complexes are formed and CaM C-lobe binds first to CaM-binding domain, then CaM N-lobe can access its site and provoke the detachment of PMCA C-terminal part from the catalytic domain, enhancing catalytic activity. KCNN4 is a homotetramer, in which gating is controlled by CaM (56, 57, 58, 59). Each KCNN4 monomer is constitutively bound to CaM C-lobe. Ca^2+^ binding to CaM induces a conformational change with the N-lobe moving toward an adjacent channel monomer, and each CaM crosslinks two adjacent KCNN4 monomers, which in turn opens the channel. A role of CaM as an intermediate between PMCA4b and KCNN4 activity was tempting. However, CaM interaction with PMCA4b is driven by its C-lobe at first, and the constitutively KCNN4-bound CaM only exposes a free N-lobe. These specific molecular interactions rule out a role of CaM in the inhibitory mechanism between PMCA4b and KCNN4.

We did not identify the PMCA4b structural domain(s) involved in KCNN4 IP, linking either directly or not the two proteins. This interaction did not impair KCNN4 activity. It could be proposed that the site(s) of interaction between KCNN4 and PMCA4b involve(s) the nucleotide-binding domain of PMCA4b that has been shown to interact with several proteins (60). Further work will be needed to define the structural domains that are interacting with each other in KCNN4 and PMCA4b.

PMCA4b-dependent regulation was not restricted to KCNN4 but can be extended to KCNN2. This enlarges the possible physiological consequences of this regulation notably in hippocampal neurons where both proteins are expressed (61). In cancer cells, KCNN2 and KCNN4 activity triggers their migration (3, 24, 62). PMCA4b expression is decreased in some cancer cells promoting migration and metastasis (63). The mechanism of reduced Ca^2+^-dependent K^+^ channel activity by interaction with PMCA4b we described could contribute to control the prometastatic role of these channels in cancer cells. This is sustained by the observation that the oncogenic activity of breast tumor cells is increased by the activation of ion channel Orai1 through a molecular interaction with the Ca^2+^ pump secretory pathway Ca^2+^ ATPase (64).

Going back to RBC where the control of KCNN4 activity is crucial for water homeostasis, PMCA4b appears as a new partner in the functional coupling between PIEZO1 and KCNN4.

**Figure 8:**
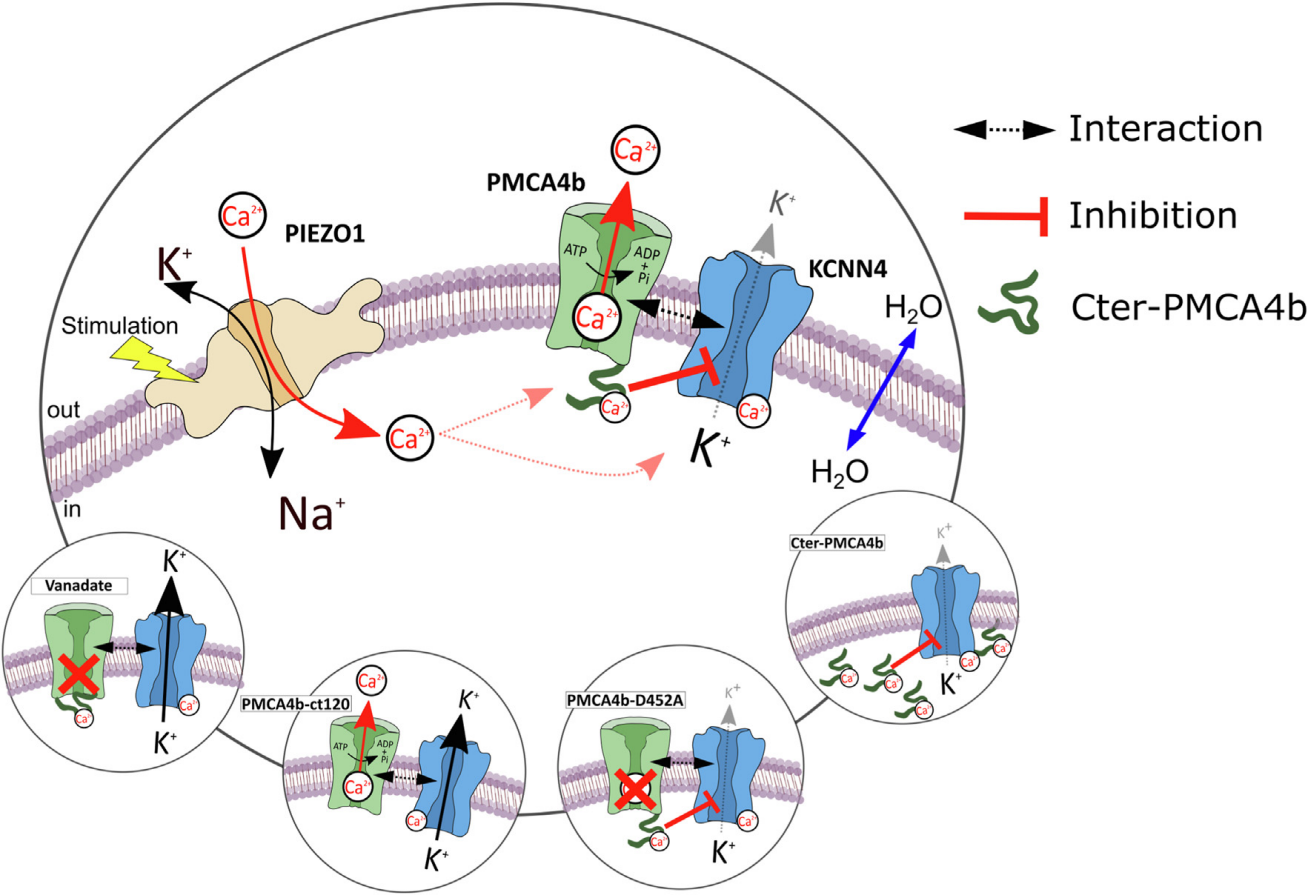
**Recapitulating schema**.

The activity of KCNN4 is dependent on the activation of ion channels permeable to Ca^2+^ such as PIEZO1, which increase free Ca^2+^ availability. By decreasing the Ca^2+^ availability, the Ca^2+^ pump reduces KCNN4 activity. However, it could also inhibit KCNN4 through a molecular interaction that is dependent on Ca^2+^ pump conformation. Thus, there is a three-member partnership in RBC membrane between ion channels permeable to Ca^2+^, KCNN4, and PMCA4b that controls RBC dehydration (Fig. 8). Recent studies have shown a link between *ATP2B4* polymorphism and RBC hydration status. In mice knockdown for *atp2b4*, RBCs have increased hemoglobin concentration (greater mean corpuscular hemoglobin concentration) compared with WT mice. This could indicate a dehydration because of a loss of control by the calcium pump on KCNN4 in addition to the observed Ca^2+^ increase in these RBCs (65). Moreover, a minor haplotype in *ATP2B4* associated with reduced PMCA4b expression in human RBC is also correlated to increased mean corpuscular hemoglobin concentration suggesting a greater KCNN4 activity in these cells (42). Further knowledge on this interaction would improve our understanding of the Gardos effect and could provide new target to treat RBC disorders linked to dehydration.

## Experimental procedures

### Red blood cells

Fresh venous blood was obtained by venipuncture in EDTA-collecting tubes from healthy volunteers who were informed and consent. The procedure was done according to the latest declaration of Helsinki (2013) and registered by the Ethic committee CPP Île de France (GR-Ex/N° DC-2016–2618).

### Na^+^, K^+^, and water content measurements

The experiments were carried out as already published (66). Blood was washed four times (800 g, 5 min, 4°C, swinging rotor) in Ringer medium containing (millimolar): NaCl (145) KCl (5), MgSO_4_ (2), CaCl_2_ (1), and Hepes–NaOH (pH 7.4) (10). RBC suspension was used at room temperature and 25% hematocrit with 0.1 mM ouabain. Na^+^ and K^+^ were quantified by flame spectroscopy with a Solaar AA spectrometer. Na_3_VO_4_ (Sigma) was solubilized at 100 mM in Milli-Q water (pH 10) adjusted with HCl, heated until clear, and cooled. Yoda1, Ouabain, 4Br-A23187, and Senicapoc were prepared in dimethyl sulfoxide stock solutions 1000 times concentrated.

### Ca^2+^ measurements

Four microliters of RBC washed in Ringer without Ca^2+^ were loaded with 2.5 μl of 1 mM Fluo4 AM stock solution in 500 μl Ringer without Ca^2+^, 37°C, 30 min. Twenty-five microliters of Fluo4=loaded RBC suspension in 975 μl Ringer medium were used to quantify relative changes in free intracellular Ca^2+^ concentrations with an FACS Fortessa BD. A total of 10,000 events were analyzed, and the whole population of RBC was considered (Fig. S5). Internal RBC fluorescence was assessed on RBC treated without Fluo4 AM. Data are expressed as a ratio of mean internal fluorescence after the addition of drug (IF) *versus* mean internal fluorescence at time 0 before the addition of drug (IF_0_). This presentation enabled to compare RBC samples loaded independently. The mean IF_0_ ± SD was 413 ± 89 arbitrary units.

### Plasmid constructs

pcDNA3-hKCNN4-HA was a kind gift of Len Kaczmarek laboratory. pcDNA3-hKCNN2 was a kind gift from Neil V. Marrion, Bristol University. peGFP-hPMCA4b and peGFP-hPMCA4bct120 were a gift from Emanuel Strehler (Addgene plasmid #47589; http://n2t.net/addgene:47589; Research Resource Identifier: Addgene_47589 and Addgene plasmid #47593; http://n2t.net/addgene:47593; Research Resource Identifier: Addgene_47593). Three single-point mutations had been detected in peGFP-hPMCA4b and hPMCA4bct120, two of them leading to amino acid substitution (c:2675A > G, p:K442R and c:3368 C > T, p:P673L). These two mutations were corrected by two successive PCR on the plasmids deposited in Addgene with oligonucleotides overlapping 15 nucleotides upstream and downstream the wrong base using KAPA high-fidelity polymerase kit (Roche). Point mutation D452A was done by PCR on peGFP-hPMCA4b (corrected for mutations on the Addgene plasmid) with the proofreading KAPA high-fidelity polymerase and primers covering 16 nucleotides upstream and downstream Asp452. CTer PMCA4b-HA was constructed by amplifying with KAPA high fidelity the 120 C-terminal amino acids of peGFP-PMCA4b with forward primer: AAGTGGTACCATGCTGCGCCGAGGCCAGAT introducing a KpnI restriction site and reverse primer: CTAGCTCGAGAACTGATGTCTCTAGGCTCT introducing an HA tag and XhoI restriction site. The PCR product was cloned in pcDNA3 KpnI–XhoI. All constructs were sequenced to check for correct DNA sequences.

### Cell transfection

HEK293T cells (purchased from American Type Culture Collection) were grown in Dulbecco's modified Eagle's medium glutamax (Gibco) and 10% fetal bovine serum and penicillin–streptomycin. Cells at ∼70% confluence were transfected using CaPO_4_ with 1 μg of plasmid DNA per milliliter of culture medium (0.5 + 0.5 μg of plasmid DNA in case of cotransfection). Sixteen hours later, cells were washed twice with PBS. For patch-clamp experiments, peGFP (plasmid coding for the GFP) was cotransfected with constructs that did not carry fluorescent tag to select fluorescent cells as transfected cells.

K562 cells were grown in RPMI1640 10% fetal bovine serum and penicillin–streptomycin. They were transfected with peGFP (control) or Cter PMCA4b using Ingenio electroporation solution and Ingenio EZporator electroporation system from Mirus Bio (10^6^ cells transfected in 100 μl solution with 2 μg DNA). Few hours before electrophysiology measurements, they were seeded on polylysine-coated plates (≈20,000 cells/ml).

### Protein expression assay

Twenty-four hours after transfection, HEK293T cells were biotinylated following the manufacturer's instructions (Pierce cell surface protein assay) and then lysed on ice, 30 min, radioimmunoprecipitation assay buffer (pH 8) with protease inhibitors (cOmplete tablets; Roche). The lysate was loaded on avidin–agarose beads. Total fraction and biotinylated fraction were subjected to SDS-PAGE and Western blot. Immunolabeling was done using primary antibodies: anti-KCNN4 (Proteintech; #23271-1-AP against peptide 128-427; rabbit, 1:1000 dilution), anti-pan-PMCA 5F10 (against peptide 724 to 783 of human RBC Ca^2+^ ATPase; Invitrogen, mouse, 1:1000 dilution), and anti-ECadherin (mouse; BD, 1:5000 dilution) for 1 h and 30 min at room temperature in TBS–Tween 0.1% to 5% low-fat milk. Horseradish peroxidase–coupled secondary antibodies were incubated for 50 min at room temperature using anti-rabbit (1:2000 dilution; DAKO) and anti-mouse (1:5000 dilution; DAKO). Horseradish peroxidase–labeled proteins were revealed with Enhanced Chemiluminescent solution (Millipore) with a Fusion FX EDGE. Quantification of band intensity was made with ImageJ software (https://imageJ.net/ij/).

### Immunoprecipitation

Pierce co-IP kit was used following the manufacturer's instructions. Ten micrograms of primary antibodies were covalently bound to agarose beads. Empty beads or beads coupled to mouse IgG (IgG2b Enzo Life Sciences) were used as control without any difference. It was thus decided to use empty beads for negative control IP experiments. HEK293T cells were lysed in IP-lysis buffer with protease inhibitors (cOmplete tablets), and the lysate was precleared on dedicated agarose beads. Primary antibodies were anti-KCNN4 (1:1000 dilution; Proteintech, #23271-1-AP, rabbit); anti-KCNN2 (Alomone rabbit against peptide 542 to 599 of rat KCNN2); anti-GFP (1:1000 dilution; Cell Signaling, rabbit), or anti-PMCA (1:1000 dilution; 5F10 monoclonal mouse; Invitrogen). One milligram of cleared lysate was loaded on immunoglobulin beads or control beads and incubated overnight at 4°C under gentle shaking. For IP with vanadate, 5 mM vanadate was added to cell lysate during IP with agarose-IgG-coupled beads overnight at 4°C. The elution (50 μl) was subjected to SDS-PAGE and Western blot.

### Proximity ligation assay

The DuoLink PLA (Olink Bioscience) was used to detect interaction between KCNN4 and Ca^2+^ pump in HEK293T cells and RBC. HEK293T cells were transfected or not with pEGFP-hPMCA4b and/or pcDNA3-hKCNN4-HA (0.5 μg DNA/ml of Dulbecco's modified Eagle's medium) using CaPO_4_ during 8 h and then seeded on polylysine (40 μg/ml)-coated microscope slides and grown until 70% confluency. Cells were washed with 1X PBS, fixed with 4% paraformaldehyde for 10 min, permeabilized for 10 min with Triton X-100 (0.03%). Blocking of unspecific antigen was done with blocking solution from kit during 1 h at 37°C. Cells were immunolabeled with primary antibodies anti-KCNN4 (1:200 dilution; rabbit; Proteintech) and anti-PMCA4b (1:200 dilution, mouse; JA3 Millipore) in kit solution overnight at 4°C. These antibodies detected KCNN4 and PMCA on HEK293T-transfected cells or RBC in immunofluorescence assays (Fig. S4).

Next steps were done according to the manufacturer's instruction (Duolink). For RBC, fresh blood drawn was washed in Ringer without Ca^2+^. Then, 20 μl of pellet was fixed for 30 min at room temperature in 1 ml 1X PBS containing 4% paraformaldehyde and 0.05% glutaraldehyde. Fixed RBCs were stored at 4°C until use. PLA was done in suspension with 20 μl of previously fixed RBC. RBCs were immunolabeled with primary antibodies anti-KCNN4 (1:200 dilution, rabbit; Proteintech) and anti-panPMCA (1:200 dilution; mouse; Invitrogen) in kit solution overnight at 4°C. Except for centrifugation steps (1 min at 1000 rpm) to pellet the cells and remove supernatant, the rest was done according to the manufacturer’s instruction in the same conditions as HEK293T cells. PLA images were captured using an inverted Zeiss Axio Observer Z1 microscope (Zeiss) x64, Cyan5 filter for PLA dot (far red), and 4′,6-diamidino-2-phenylindole filter for nucleus. Confocal L880 Zeiss microscope, x64 were used for illustration. For quantification, PLA dots per cells were quantified using ImageJ software and compared with control condition: nontransfected HEK293T cells or RBC with only secondary antibodies.

### Electrophysiology

Glass pipettes (Brand) with final resistance of 3 to 5 MΩ were made on a horizontal pipette puller (P-97; Sutter Instrument). All patch-clamp experiments were performed with a PC-controlled EPC9 patch-clamp amplifier (HEKA; Lambrecht/Pfalz). Currents were acquired and analyzed with Pulse and PulseFit software (HEKA).

Currents were measured in whole-cell configuration with bath solution in millimolar: 150 NaCl, 5 KCl, 1 CaCl_2_, 1 MgCl_2_, 10 Hepes at pH 7.4 and inside: 150 KCl, 5 NaCl, 1 MgCl_2,_ 1 EGTA, 10 Hepes, 0.03 CaCl_2_ to reach 10 nM of free Ca^2+^ at pH 7.2. Free Ca^2+^ concentrations were adjusted by adding CaCl_2_ using Ca–EGTA Calculator v1.3. Currents were measured at room temperature using a ramp protocol from −120 to +80 mV from a holding potential of −60 mV (sampling frequency 10 kHz; filtered 5 kHz).

### Statistical analysis

GraphPad Prism 9 (GraphPad Software, Inc) was used for statistical analyses. For one comparison, Man–Whitney test, for multiple comparisons, ANOVA or Kruskall–Wallis test were applied with a post hoc Dunn’s correction when comparing each condition or uncorrected Dunn’s test when comparing only against one condition. For grouped analysis, two-way ANOVA with Dunnet’s post hoc or Tukey correction was used.

## Data availability

All data are contained in the article and supporting information. The authors require that any published work derived from the use of these data include a reference to this publication.

## Supporting information

This article contains supporting information.

## Conflict of interest

The authors declare that they have no conflicts of interest with the contents of this article.
