# Supplementary material for: A new regulation mechanism for KCNN4, the Ca2+-dependent K+ channel, by molecular interactions with the Ca2+pump PMCA4b
Source: J Biol Chem. 2024 Dec 21;301(2):108114. doi: 10.1016/j.jbc.2024.108114 (PMC11787511; doi:10.1016/j.jbc.2024.108114)
Supplement: Supplemental Table S1 and S2 and Figures S1-S5 [file mmc1.docx]

**Supporting information**

“A new regulation mechanism for KCNN4, the Ca^2+^-dependent K^+^ channel, by molecular interactions with the Ca^2+^pump PMCA4b”

Benoit Allegrini, Morgane Mignotet, Raphaël Rapetti-Mauss, Franck Borgese, Olivier Soriani, Hélène Guizouarn

-Supplemental methods (1)

-Supplemental tables (2)

-Supplemental figures (5)

**Supplemental Methods:**

Ca^2+^measurements:

To avoid any changes in intracellular water content while measuring Ca^2+^ associated fluorescence in presence of Ca^2+^ ionophore 4-Br-A23187 experiments were carried on in high K^+^ medium (90 mM KCl+ 55 mM NaCl instead of 145 mM NaCl in Ringer solution) to achieve electro-chemical equilibrium for K^+^. The opening of KCNN4 in this medium did not lead to net K^+^ movement and there was no dehydration that could alter Ca^2+^ concentration measurements. The same control experiment was done with RBC stimulated by Yoda1 +/- vanadate to control that Fluo4 fluorescence was similar in high K^+^ medium and Ringer medium. In addition, it was checked that vanadate or Yoda1 addition did not significantly modify endogenous RBC fluorescence by doing the experiments in Ringer without Ca^2+^.

**- Supplemental table 1:**

| Mean±S.D. | Na^+^ | K^+^ | Na^+^+K^+^ | ∆Na^+^ | ∆K^+^ | Ca^2+^ IF/IF_0_ |
| --- | --- | --- | --- | --- | --- | --- |
| t0 | 24.7±14.4 | 225.1±14.0 | 249.8±23.2 | 0 | 0 | 1 |
| Yoda1 20 min | 47.7±22.8 | 191.2±43.5 | 239.1±21.8 | 23.0±14.3 | -34.4±18.6 | 14.6±4.0 |
| Yoda1+Senicapoc 20 min | 56.6±11.3 | 195.4±18.4 | 251.2±16.3 | 36.5±9.3 | -34.4±13.9 |  |
| Yoda1 40 min | 65.0±21.0 | 160.9±14.3 | 225.9±16.0^**^ | 46.0±22.5 | -83.8±14.3^*^ | 13.4±4.1 |
| Yoda1+Senicapoc 40 min | 74.2±13.3 | 175.0±16.0 | 245.7±10.7 | 59.0±11.3 | -56.5±13.5 |  |
| Yoda1+vanadate 20 min | 43.0±14.3 | 157.7±15.3 | 200.7±18.9^****^ | 21.1±9.1 | -66.9±19^****^ | 22.2±2.8 |
| Yoda1+vanadate+Senicapoc 20 min | 47.1±27.0 | 197.0±15.6 | 240.7±17.9 | 25.7±16.5 | -18.1±13 |  |
| Yoda1 + vanadate 40 min | 41.1±20.6 | 116.8±15.8 | 168.8±18.7^****^ | 40.4±22.6 | -117.0±21.9^****^ | 23.2±2.6 |
| Yoda1+vanadate+Senicapoc 40 min | 50.1±16.0 | 189.0±2.0 | 239.1±14.0 | 30.9±9.8 | -46.0±0.6 |  |
| Vanadate 20 min | 25.1±10.6 | 216.2±14.7 | 240.1±17.9 | 1.2±7.6 | -8.7±15.1 | 4.2±3.4 |
| Vanadate +Senicapoc 20 min | 18.4±3.2 | 206.5±19.3 | 235.8±22.1 | 0.6±0.0 | -7.9±11.4 |  |
| Vanadate 40 min | 27.6±11.5 | 189.2±23.1 | 215.2±25.6^****^ | 0.7±13.1 | -37.5±28.0^****^ | 8.1±4.0 |
| Vanadate+Senicapoc 40 min | 21.2±10.6 | 221.9±7.5 | 240.9±17.8 | -2.1±8.9 | -7.8±17.0 |  |

Means±S.D. of Na^+^ and K^+^ contents in µmol/g dry weight used to draw figure 1. n=26 for t=0, n=22 for Yoda1, n=5 for Yoda1+Senicapoc, n=15 for Yoda1+vanadate, n=3 for Yoda1+vanadate+Senicapoc, n=20 for vanadate, n=14 and n=2 for vanadate+Senicapoc (40’ and 20’ respectively). For Ca^2+^ n=17 and n=11 for Yoda (20’ and 40’ respectively), n=10 and n=5 Yoda+vanadate (20’ and 40’ respectively), n=18 and n=13 for vanadate (20’ and 40’ respectively). The sum (Na^+^+K^+^) and the variations between t=0 and t=20 or 40 min. for Na^+^ and for K^+^ contents were calculated for each individual experiment. A Two-way ANOVA with Tukey’s multiple comparisons test was done to compare (Na^+^ +K^+^) in different conditions with control condition t=0 and to compare ∆Na^+^ and ⎜∆K^+^⎜ in the same conditions. **** p<0.0001, **p≤0.05, * p=0.06.

**- Supplemental table 2:**

| time min | Yoda1 2µM | Yoda1 Bum. 0.1 mM | Yoda1 Vana 1 mM | Yoda1 Vana Bum | Vana 1 mM | Vana Bum.0.1 mM |
| --- | --- | --- | --- | --- | --- | --- |
| 0 | 234.5±13.9 | 234.5±13.9 | 234.5±13.9 | 234.5±13.9 | 234.5±13.9 | 234.5±13.9 |
| 5 | 208.3±6.2 | 204.9±11.0 | 197.6±7.0 |  |  |  |
| 20 | 188.6±6.5 | 194.6±2.2 | 147.4±1.8 |  | 221.8±5.5 |  |
| 40 | 168.8±1.4 | 172.5±3.8 | 105.6±1.8 | 111.8±5.7 | 205.0±4.2 | 203.5±1.8 |

Means±S.D (n=3) of K^+^ contents in RBC before (t=0) and at different time after treatment by 2 µM Yoda1 ± 1 mM vanadate ± 0.1 mM bumetanide. There was no significant difference in presence or absence of bumetanide.

**- Supplemental figure 1**: Effect of 1 mM versus 5 mM vanadate on the apparent intracellular Ca^2+^ concentration in RBC.

Same experiment as shown in figure 1D. One representative experiment over 2.

**- Supplemental figure 2:** Effect of Vanadate on KCNN4 activity in HEK293T transfected cells.


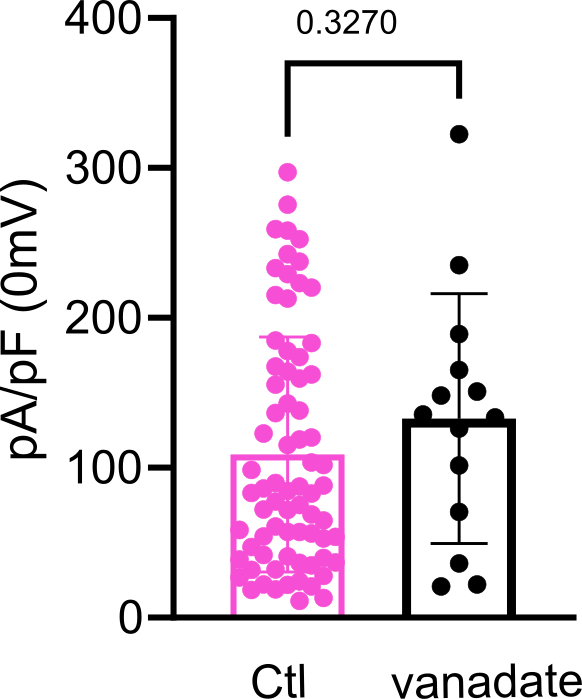


HEK293T cells were transfected as in figure 4 with KCNN4. Currents were recorded in absence (pink) or presence (black) of vanadate incubated for 20 minutes in extracellular bath, pCai=6. Figure shows current density in pA/pF at 0 mV, means±S.D. n=73 cells for control condition, n=14 cells for vanadate condition. Data were analyzed with Mann-Wittney test.

**- Supplemental figure 3:** Effect of Senicapoc on endogenous K562 currents.

Control, no Fluo4, 8176 single cells

Yoda1, t=0, no Fluo4 9916 single cells

Vanadate t=0 min, 7477 single cells

Yoda1 t=0 min, 9617 single cells

Yoda1 t=20 min, 7773 single cells

Yoda1 t=30 min, 9758 single cells

Control t=0 min, 8214 single cells

Yoda1+ Vanadate t=0 min, 9274 single cells

Yoda1+ Vanadate t=20 min, 9703 single cells

Yoda1+ Vanadate t=30 min, 9717 single cells

Yoda1+ Vanadate t=10 min, 9659 single cells

Vanadate t=10 min, 7955 single cells

Vanadate t=20 min, 8851 single cells

Vanadate t=30 min, 9688 single cells


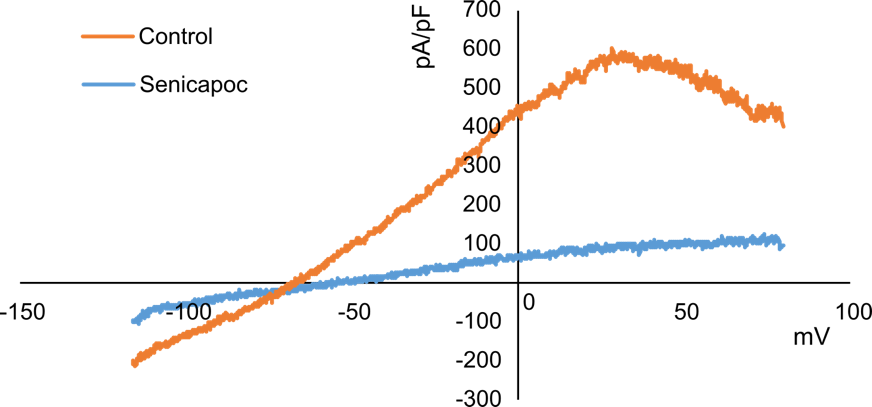


Representative traces of current in K562 cells, control (orange) or treated by 300 nM Senicapoc (blue), pCai=5. The mean inhibition ± S.D. was 65±19 %, n=3.

- **Supplemental figure 4**: Effect of Dab7, specific inhibitor of KCNN2


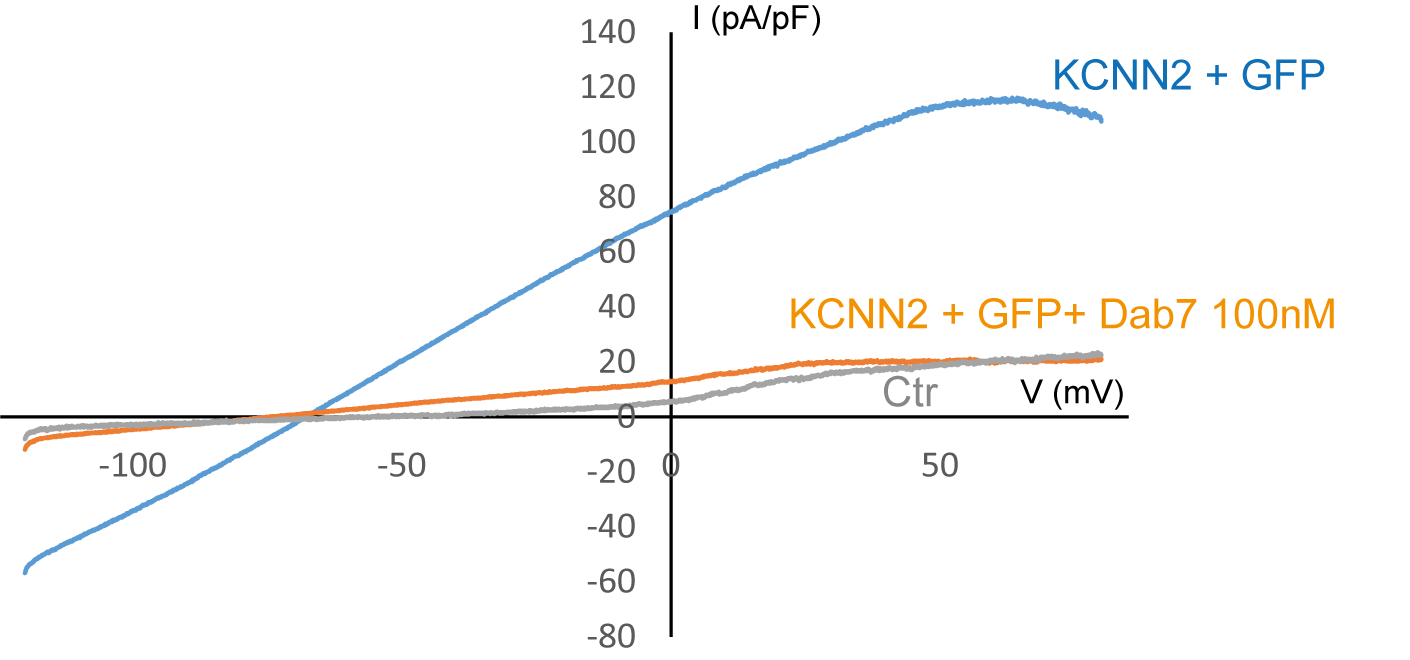


HEK293T cells were transfected as in figure 7. Representative traces of currents recorded in control HEK293T cells (grey line) and HEK293T cells co-transfected with KCNN2 and GFP without (blue line) or with100 nM Dab7 in the pipette (orange line), pCai=6. The mean inhibition was 76% (n=3).

- **Supplemental figure 5**: Histograms showing the fluorescent signal associated with RBC in different experimental conditions at different time.


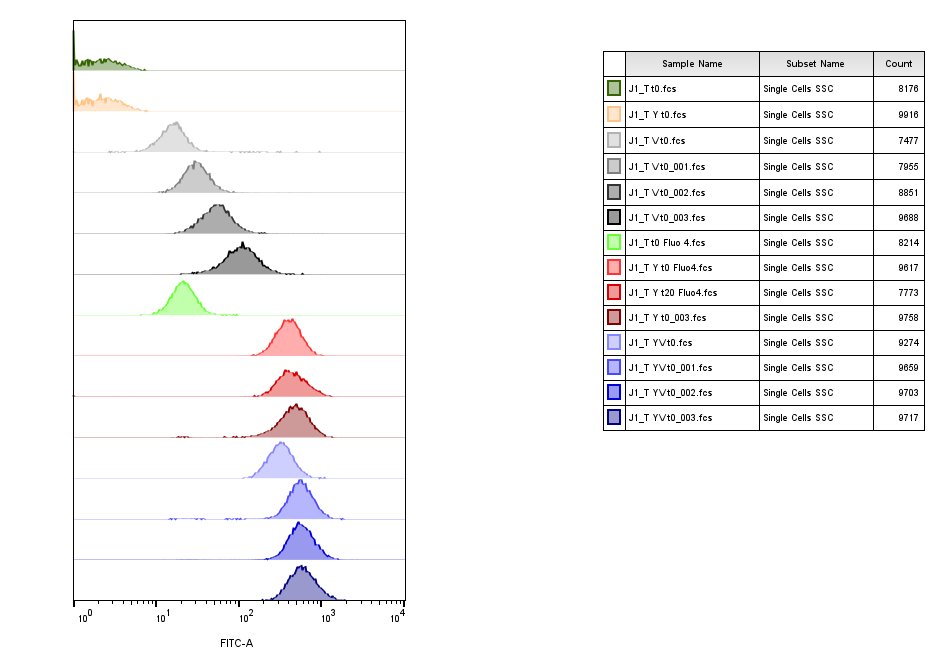


Control, no Fluo4, 8176 single cells

Yoda1, t=0, no Fluo4 9916 single cells

Vanadate t=0 min, 7477 single cells

Yoda1 t=0 min, 9617 single cells

Yoda1 t=20 min, 7773 single cells

Yoda1 t=30 min, 9758 single cells

Control t=0 min, 8214 single cells

Yoda1+ Vanadate t=0 min, 9274 single cells

Yoda1+ Vanadate t=20 min, 9703 single cells

Yoda1+ Vanadate t=30 min, 9717 single cells

Yoda1+ Vanadate t=10 min, 9659 single cells

Vanadate t=10 min, 7955 single cells

Vanadate t=20 min, 8851 single cells

Vanadate t=30 min, 9688 single cells

Cell number

t=0 min corresponded to a measurement made about 5 seconds after the addition of drug to RBC sample. The same RBC sample was then measured at indicated time. The figure was drawn with the cytometry software FlowJo on one representative experiment. Except for the two first conditions, there was Fluo4 in all conditions.
